# A Comprehensive Evaluation Method of Sensor Selection for PHM Based on Grey Clustering

**DOI:** 10.3390/s20061710

**Published:** 2020-03-19

**Authors:** Fei Guan, Wei-Wei Cui, Lian-Feng Li, Jie Wu

**Affiliations:** 1 China Academy of Launch Vehicle Technology, Beijing 100076, China; buaacvv@126.com (W.-W.C.);; 2 College of Aerospace Science and Engineering, National University of Defense Technology, Changsha 410073, China; nudt_wujie@126.com

**Keywords:** sensor selection and evaluation, PHM, grey clustering, dependency matrix, whitening weight function

## Abstract

Sensor selection plays an essential and fundamental role in prognostics and health management technology, and it is closely related to fault diagnosis, life prediction, and health assessment. The existing methods of sensor selection do not have an evaluation standard, which leads to different selection results. It is not helpful for the selection and layout of sensors. This paper proposes a comprehensive evaluation method of sensor selection for prognostics and health management (PHM) based on grey clustering. The described approach divides sensors into three grey classes, and defines and quantifies three grey indexes based on a dependency matrix. After a brief introduction to the whitening weight function, we propose a combination weight considering the objective data and subjective tendency to improve the effectiveness of the selection result. Finally, the clustering result of sensors is obtained by analyzing the clustering coefficient, which is calculated based on the grey clustering theory. The proposed approach is illustrated by an electronic control system, in which the effectiveness of different methods of sensor selection is compared. The result shows that the technique can give a convincing analysis result by evaluating the selection results of different methods, and is also very helpful for adjusting sensors to provide a more precise result. This approach can be utilized in sensor selection and evaluation for prognostics and health management.

## 1. Introduction

With the rapid development of industrial technology, the complexity of engineering systems has increased significantly [1,2,3,4]. The increase in the failure cost of the system prompts people to pay more attention to the reliability and safety of the system [5]. Therefore, prognostics and health management (PHM) technology are widely studied and applied in the industry [3,4,6]. PHM is a method that allows for an assessment of the reliability of a system under actual application conditions. It uses the sensors to monitor the health status of a system for fault diagnosis, life prediction, and condition-based maintenance, which guides the decision making and reduces the usage and maintenance costs [7,8].

The sensor plays a significant role in PHM technology [7,9,10,11]. It is the basis of data for state evaluation and life prediction, and its selection result has a direct influence on the application effect of PHM [12]. Therefore, it is vital to optimize sensor selection to reduce the scale of sensors and obtain the same or an even better monitoring effect. The existing sensor selection methods can be grouped into two categories: the model-based method [10,13,14,15,16] and the optimization method considering testability constraints [17,18]. The former establishes a graph or mathematical model to describe the relationship between the sensors and failure modes, such as the dependency model, heuristic graph, and state diagram. Based on the models, the sensor selection results can be output through automatic traversal or matrix operation. The procedure of the later can be summarized in four steps: (1) determining an optimization goal (cost or time minimization); (2) defining testability indexes to describe testability requirement; (3) constructing the mathematical relationship between the goal and testability indexes; (4) using or designing an algorithm to solve the optimization problem under the constraints of the testability indexes. Usually, the testability indexes contain a fault detectable rate, a critical fault detectable failure rate, a fault isolation rate, and a fault prognostic rate [18,19,20]. The heuristic algorithm is commonly used to solve the optimization problem, such as particle swarm and genetic algorithm [20,21].

However, a common weakness of the existing methods is that these methods do not have a standard to select sensors, which leads to a hard choice, facing the different selection results given by these methods. In the model-based way, it cannot give a proper selection for the equivalent sensors, which have the same testing response. In the optimization way, the parameter setting of the heuristic algorithm has a vital effect on the optimization, resulting in an unstable selection result [22]. Meanwhile, the mathematical relationship is hard to be constructed because different types of parameters are difficult to be unified and processed. Therefore, it is necessary to form a principle to evaluate sensor selection of different methods, which helps to choose a better result by contrast and even adjust sensors based on the evaluation result to give a more convincing and reasonable result. 

In this paper, we propose a comprehensive evaluation method of sensor selection for PHM based on grey clustering. This method is dedicated to evaluating the sensor selection according to its multiple relevant factors to give a more reasonable and convincing selection result. Sensor selection is a process full of uncertainty, and its result depends on expert advice, similar product experience, or measured data. For eliminating or reducing the uncertainty, the grey clustering is introduced and applied. Grey clustering is an important part of the grey theory, and it mainly studies the uncertainty of the system, which is suitable and active for evaluating sensor selection. Based on the method, the key factors are considered based on the previous studies, and they are quantized, manifested, and whitened, which can give a criterion for the evaluation of sensor selection. In the calculation process of grey clustering, the data in different types can be unified. The process also expands the range of evaluation indexes, which improves the comprehensiveness of evaluation result. Meanwhile, the weight in the grey clustering is extended to a combination weight considering subjective and objective factors, which is helpful to give a more valid result.

**Figure 1:**
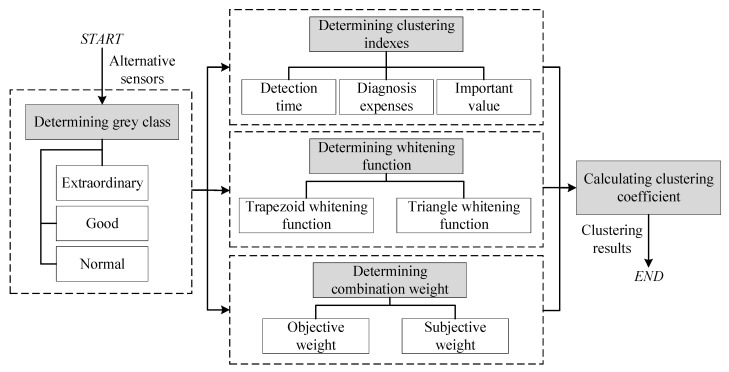
The workflow of the proposed method.

The workflow of the proposed method is shown in Figure 1. Firstly, we divide the sensors (clustering object) into three grey classes: extraordinary, good, and normal, which learns from the state division of equipment [23]. Based on the previous work on sensor selection, we determine three clustering indexes: the detection time, diagnosis expense, and importance value [15,18], which covers the factors related to sensor selection, and the indexes are quantified based on the dependency model. Then, we determine the whitening weight function to define the extent to which the clustering index belongs to a grey class, and propose a new and reasonable combination weight of the index to grey class considering the objective data and subjective factors. Finally, we obtain the sensor evaluation result by analyzing the clustering coefficients, which is calculated by the whitening weight function and the combination weight. To improve the selection result, we evaluate the selection result given by the dependency model and genetic algorithm (GA) because the two methods are commonly utilized in sensor selection. The evaluation principle is the proportion of good and extraordinary sensors. An electronic control system is taken as an example to illustrate the effectiveness of the proposed method. 

The rest of this paper is organized as follows. Section 2 gives the grey clustering model of sensors evaluation. Section 3 describes an example in which the proposed method is applied. Finally, conclusions are drawn in Section 4.

## 2. The Grey Clustering Model for Sensor Evaluation

Grey clustering is widely utilized in system analysis and assessment, and it is very good at processing multi-type parameters, which is suitable for the evaluation of sensor selection. The purpose of the proposed method is to attribute the clustering object to a grey class [24,25].

In the evaluation method based on grey clustering, there are *n* clustering objects and *m* clustering indexes. We define the sensors $SO=\{so_{1},so_{2},\ldots ,so_{n}\}$ as the clustering object, and define three grey classes: extraordinary (E), good (G), and normal (N). The grey classes are denoted as $s=\{s_{1}=E,s_{2}=G,s_{3}=N\}$. The clustering indexes are the key factors to evaluate the clustering objects, and they are denoted as $c=\{c_{1},c_{2},\ldots ,c_{m}\}$.

### 2.1. Clustering Index

It is essential to identify a fault in the design accurately and in a timely fashion for the testability of equipment [26], so the detection time and the failure rate is worth considering as the clustering indexes. Based on the previous analysis, the cost is usually taken as the objective function in the optimization method. Therefore, we define the detection time, diagnosis expense, and importance value as the clustering indexes, which is extensive and comprehensive. The detection time is used to measure the timeliness of a sensor, diagnosis expense is to measure the economic attribute, and the importance value is to describe the degree of failure mode detection by a sensor. These three indexes are defined and quantified as follows.

#### 2.1.1. Detection Time

We define the maximum value of the time that a sensor spends on the different detectable failure modes as the detection time using the following equation:

(1) $$c_{1}=\max(\Delta t_{ij})$$

where $\Delta t_{ij}$ is the detection time that the *ith* sensor spends on the *jth* failure mode.

#### 2.1.2. Diagnosis expense

We define the average expense that a test spends on the detectable failure modes as the diagnosis expense. To quantify the diagnosis expense, we introduce the dependency matrix from the dependency model. The dependency matrix describes the relationship between the sensors (SO) and failure modes (F), and it is expressed as follows:

(2) $$\begin{array}{l} \begin{array}{cccc} & SO_{1} & SO_{2} & \begin{array}{cc} \ldots & SO_{n} \end{array} \end{array}\\ D=\begin{array}{c} F_{1}\\ F_{2}\\ \ldots \\ F_{m} \end{array}\left[\begin{array}{l} d_{11}\begin{array}{ccc} d_{12} & \ldots & d_{1n} \end{array}\\ d_{21}\begin{array}{ccc} d_{22} & \ldots & d_{2n} \end{array}\\ \ldots \begin{array}{ccc} & & \begin{array}{cc} & \ldots \end{array} \end{array}\\ d_{m1}\begin{array}{ccc} d_{m2} & \ldots & d_{mn} \end{array} \end{array}\right] \end{array}$$

where *d_ij_ =* 1 if the *ith* sensor can detect the *jth* failure mode, or *d_ij_ =* 0.

Based on the dependency matrix, the diagnosis expense of a sensor is as follows:

(3) $$c_{2}=\frac{\sum_{i=1}^{m}e_{ij}}{l},i=1,2,\ldots ,l\leq m$$

where *e_ij_* is the expense that the *ith* sensor spends on *jth* detectable failure mode detection, and *l* is the number of detectable failure modes.

#### 2.1.3. Importance Value

We define the failure rates’ sum of the failure modes that a sensor can detect as the importance value. Based on the dependency matrix, the importance value is as follows:

(4) $$c_{3}=\sum_{i=1}^{m}d_{ij}\cdot \lambda_{i}$$

where $\lambda_{i}$ is the failure rate of the *ith* failure mode.

After determining the indexes, we can obtain the observation matrix of the clustering object to indexes, which can be expressed as follows:

(5) $$\begin{array}{l} \begin{array}{cccc} & c_{1} & c_{2} & \begin{array}{ccc} c_{3} & & \end{array} \end{array}\\ \begin{array}{c} SO_{1}\\ SO_{2}\\ \ldots \\ SO_{n} \end{array}\left[\begin{array}{ccc} x_{11} & x_{12} & x_{13}\\ x_{21} & x_{22} & x_{23}\\ \ldots & \ldots & \ldots \\ x_{n1} & x_{n2} & x_{n3} \end{array}\right] \end{array}$$

### 2.2. Whitening Weight Function

**Figure 2:**
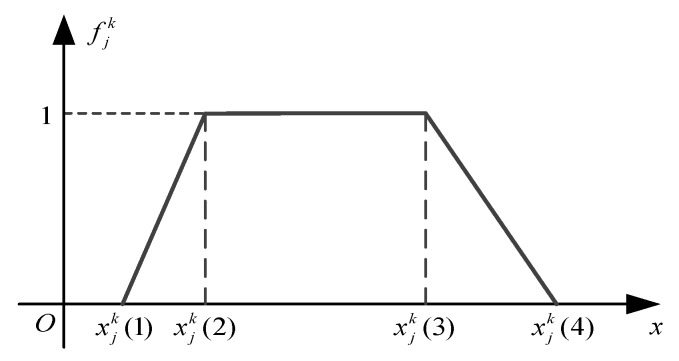
The typical whitening weight function.

The whitening weight function is utilized to describe the extent to which the clustering index belongs to a grey class. The whitening weight function of the *jth* index belonging to the *kth* grey class is denoted as $f_{j}^{k}(x_{ij})$. A typical whitening weight function is shown in Figure 2.

In Figure 2, $x_{j}^{k}(1)\text{,}x_{j}^{k}(2)\text{,}x_{j}^{k}(3)\text{,}x_{j}^{k}(4)$ are the turning points. A whitening weight function can be represented using these points [27,28], and it is expressed using the following equation:

(6) $$f_{j}^{k}(\;x_{ij}\;)=f_{j}^{k}[x_{j}^{k}(1),x_{j}^{k}(2),x_{j}^{k}(3),x_{j}^{k}(4)]$$

**Figure 3:**
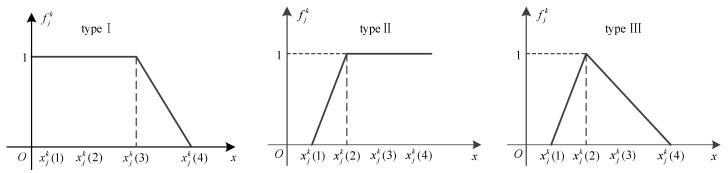
The whitening weight function types.

A whitening weight function can be divided into three types, as shown in Figure 3. They are the trapezoid whitening function (typeⅠand type Ⅱ) and the triangle whitening weight function (type Ⅲ).

It is obvious that the less detection time is helpful to improve the health assessment capability of a system, so we choose Type I as the whitening weight function of the detection time index belonging to E class. That means if the detection time of a sensor is too long, then the degree to which it belongs to E will decrease. If the value of the detection time continues to increase, its attribution will move away from E and towards G. That means the degree to which it belongs to E decreases and the degree to which it belongs to G increases. When its value is higher than a certain level, its attribution will move away from G and towards N. Therefore, we choose Type III as the whitening weight function of the index belonging to G class. Similarly, the whitening weight function’s selection of other indexes to the grey class is shown in Table 1.

### 2.3. Combination Weight

The weight η in grey clustering is utilized to calculate the clustering coefficient, and it represents the weight of the index to grey classes. The weight has a direct effect on the clustering result. The methods for weight determination contain the subjective and objective approach, and both of them have its advantages. However, the subjective method depends on the expert experience, and the data size has a direct effect on the objective method. The results given by the subjective and objective approach sometimes are different, and then both of them bring uncertainty to clustering analysis. Therefore, we propose a combination value integrating the advantages of the objective and subjective methods, which can give a more convincing and comprehensive weight. The combination weight includes the objective weight and subjective weight. The subjective weight $\eta_{s}$ is determined by the analytic hierarchy process (AHP), and the objective weight $\eta_{o}$ is determined by information entropy theory (IE). AHP is a commonly utilized subjective method, and IE is a dimensionless method, which can improve the comprehensiveness of weights. To synthesize the subjective and objective factors, we use a typical normalization method to process the product of the weights, and it can be expressed as follows:

(7) $$\eta ={\left[\eta_{i}\right]}_{1\times m}=\frac{\eta_{si}\cdot \eta_{oi}}{\sum_{i=1}^{m}\eta_{si}\cdot \eta_{oi}}$$

The objective weight $\eta_{o}$ is obtained by the observation matrix of the clustering object to the indexes, and the matrix is as follows:

(8) $$\begin{array}{l} \begin{array}{ccccc} & & & c_{1} & \begin{array}{ccc} c_{2} & \ldots & c_{m} \end{array} \end{array}\\ \begin{array}{c} t_{1}\\ t_{2}\\ \ldots \\ t_{n} \end{array}\left[\begin{array}{ccc} x_{11} & x_{12} & x_{1m}\\ x_{21} & x_{22} & x_{2m}\\ \ldots & & \ldots \\ x_{n1} & x_{n2} & x_{nm} \end{array}\right] \end{array}$$

Then, we calculate the objective weight as follows [29]:

(9) $$\left\{ \begin{array}{l} \eta_{oi}=\frac{p_{i}}{\sum_{i=1}^{m}p_{i}},i=1,2,\ldots ,m\\ p_{i}=1-h_{i},i=1,2,\ldots ,m\\ h_{i}=-k\sum_{i=1}^{n}f_{ij}\ln f_{ij},k=1/\ln n\\ f_{ij}=x_{ij}/\sum_{i=1}^{n}x_{ij} \end{array}\right.$$

We can obtain the subjective weight $\eta_{s}$ by a judgment matrix, and the matrix is as follows:

(10) $$\begin{array}{l} \begin{array}{cccc} & & & \begin{array}{cccc} c_{1} & c_{2} & \ldots & c_{m} \end{array} \end{array}\\ \begin{array}{c} c_{1}\\ c_{2}\\ \ldots \\ c_{4} \end{array}\left[\begin{array}{l} a_{11},a_{12},\ldots ,a_{1m}\\ a_{21},a_{22},\ldots ,a_{2m}\\ \ldots \\ a_{m1},a_{m2},\ldots ,a_{mm} \end{array}\right] \end{array}$$

where $a_{ij}$ is determined as shown in Table 2.

Based on the judgment matrix, we can calculate the subjective weight as follows [30]:

(11) $$\left\{ \begin{array}{l} \eta_{si}=\frac{p_{i}}{\sum_{i=1}^{m}p_{i}},i=1,2,\ldots ,m\\ p_{i}=\sum_{j=1}^{m}b_{ij},i=1,2,\ldots ,m\\ b_{ij}=a_{ij}/\sum_{i=1}^{m}a_{ij}\\ a_{ij}\cdot a_{ji}=1 \end{array}\right.$$

### 2.4. Clustering Coefficient and Clustering Result

For every clustering object, the clustering coefficient $\sigma_{i}^{k}$ is utilized to measure the degree of the *ith* object belonging to the *kth* grey class, and it is as follows:

(12) $$\sigma_{i}^{k}=\sum_{j=1}^{m}f_{j}^{k}(x_{ij})\cdot \eta_{j},i=1,2,\ldots ,n$$

For the entire clustering objects, we can obtain a coefficient matrix, which can be expressed as follows:

(13) $$(J^{k}{}_{i})=\left[\begin{array}{l} \sigma_{1}^{1},\sigma_{1}^{2},\ldots ,\sigma_{1}^{s}\\ \sigma_{2}^{1},\sigma_{2}^{2},\ldots ,\sigma_{2}^{s}\\ \ldots \\ \sigma_{n}^{1},\sigma_{n}^{2},\ldots ,\sigma_{n}^{s} \end{array}\right]$$

In the matrix, every row is a coefficient vector. The grey class with the maximum coefficient in every vector is judged as the class of its corresponding object.

## 3. Case Study

**Figure 4:**
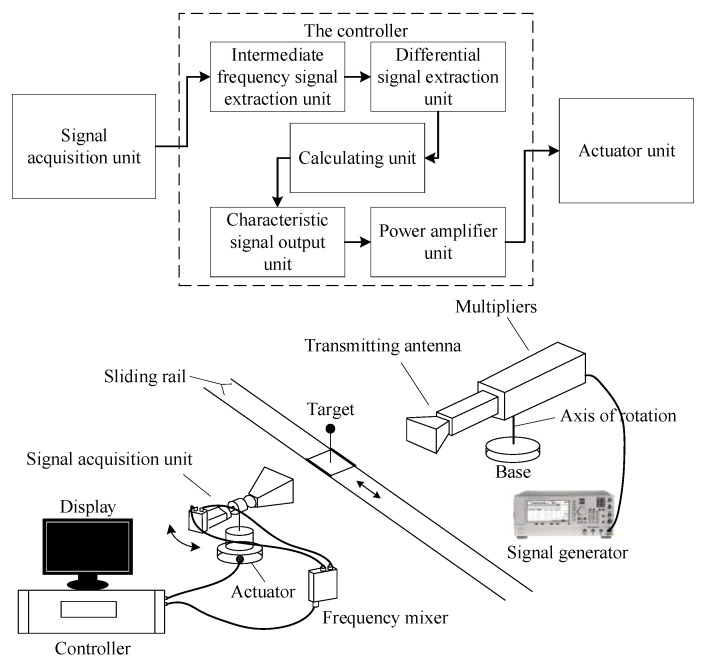
The composition of radar prototype.

To illustrate the effectiveness of the proposed method, we take an electronic control system as an example. The system is the control part of a radar prototype, and it is designed to demonstrate the target tracking characteristics. The composition of the radar prototype is shown in Figure 4.

**Figure 5:**
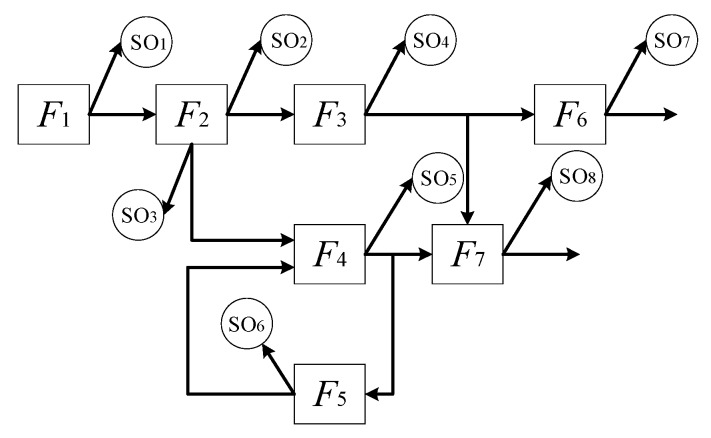
The dependency graphic model of the controller.

The target can be moved automatically on the sliding rail, and the radar receives and processes the signal to control the trace motion. The controller of the radar is a piece of complicated electronic equipment, and it has a high requirement for PHM. The failure detection rate $\gamma_{FD}$ is not less than 90%, the key failure mode $\gamma_{FDkey}$ is 100%, and the failure isolation rate $\gamma_{FI}$ is not less than 90% (ambiguity is less than 2). Based on the composition of the radar, we can obtain the dependency graphic model of the controller by the dependency model theory, which is shown in Figure 5. The dependency graphic model is used to illustrates the relationship between the failures and sensors. In the graphic model, the fault information flows with the arrows.

In Figure 5, *F* represents the failure mode, and *SO* represents the alternative sensors (voltage sensor and signal sampler). The concrete information about the failure modes and sensors is shown in Table 3.

Based on the graphic model, we obtain the dependency matrix, and it is shown in Table 4.

We obtain the detection time by circuit simulation. From the price list of the bill of material (BOM), we obtain the diagnosis expense, which is the cost of the replacement of the faulty part detected by the sensor. From the reliability prediction results based on the standards (MIL-HDBK-217F in U.S.A or GJB299C in China), we obtain the failure rates. The information is shown in Table 5, Table 6, and Table 7.

From the basic information above, we obtain the observation matrix *X* of clustering object to the clustering index. For eliminating the effect of the index dimensions, the matrix is equalized and denoted as X^*^.

(14) $$\begin{array}{l} \begin{array}{cccc} \begin{array}{cccccc} & & & & c_{1} & \end{array} & c_{2} & & c_{3} \end{array}\begin{array}{cccc} \begin{array}{cccccc} & & & & c_{1} & \end{array} & c_{2} & & c_{3} \end{array}\\ X=\begin{array}{c} T_{1}\\ T_{2}\\ T_{3}\\ T_{4}\\ T_{5}\\ T_{6}\\ T_{7}\\ T_{8} \end{array}\left[\begin{array}{ccc} 185 & 20.7 & 49.9\\ 235 & 14.2 & 54.2\\ 139 & 19.3 & 54.2\\ 294 & 13.7 & 81.7\\ 241 & 14.2 & 83.4\\ 298 & 15.5 & 83.4\\ 266 & 21.5 & 82.5\\ 269 & 9.7 & 117.7 \end{array}\right]X^{*}=\begin{array}{c} T_{1}\\ T_{2}\\ T_{3}\\ T_{4}\\ T_{5}\\ T_{6}\\ T_{7}\\ T_{8} \end{array}\left[\begin{array}{ccc} 0.77 & 1.29 & 0.66\\ 0.98 & 0.88 & 0.71\\ 0.58 & 1.20 & 0.71\\ 1.22 & 0.85 & 1.08\\ 1.00 & 0.88 & 1.10\\ 1.24 & 0.96 & 1.10\\ 1.10 & 1.34 & 1.55\\ 1.12 & 0.60 & 1.68 \end{array}\right] \end{array}$$

Then, we determine the whitening weight function, and we take the function $f_{1}^{1}$ as an example to illustrate the process. The function $f_{1}^{1}$ describes the relationship between detection time and the extraordinary class (E). Obviously, the sensors tend to the extraordinary class, as the value decreases. Therefore, we choose the type I function as the type of the detection time. The determination of the turning points mainly relies on historical data and subjective adjustments. In the historical records of testing and maintenance, there are many effect evaluations (very good, good, and normal) for every testing and maintenance activity of the operators. Based on the records, we count the range of the detection time on different evaluation levels, and then we slightly adjust the range by experience. The purpose of the adjustment is to make the different whitening functions of the index intersect because the attribution of the index is a continuous transition state, not a hard separation. The adjustment range is usually no more than 0.1 by experience. Thus, we finally obtain the turning points and the whitening weight function. Similarly, other function results are determined, as shown in Table 8.

In the following equation, we obtain the subjective weight $\eta_{s}$ by subjective assessment. The judgment matrix is as follows:

(15) $$\left[\begin{array}{ccc} 1 & 3 & 1/3\\ 1/3 & 1 & 1\\ 3 & 1 & 1 \end{array}\right]$$

Based on the previous mathematical analysis, we obtain the objective weight $\eta_{o}$ from Equations (8) and (9), and the subjective weight $\eta_{s}$ from Equations (10) and (11). Thus, we obtain the combination weight η using subjective and objective weight from Equation (7). The weights are as follows:

(16) $$\begin{array}{l} \eta_{s}=[0.3245,0.2352,0.4403]\\ \eta_{o}=[0.3417,0.3364,0.3219]\\ \eta =[0.3343,0.2385,0.4272] \end{array}$$

Finally, we obtain the coefficient matrix from Equations (12) and (13), and the matrix is as follows:

(17) $$J^{k}{}_{i}=\left[\begin{array}{l} 0.0869,\underline{0.4077},0.2990\\ 0.0119,\underline{0.3194},0.3194\\ 0.2140,\underline{0.4597},0.1922\\ 0.0982,0.0795,\underline{0.2266}\\ 0.0974,\underline{0.1272},0.1272\\ 0.0854,0.2226,\underline{0.3831}\\ \underline{0.0769},0.0000,0.0669\\ \underline{0.6060},0.0000,0.0802 \end{array}\right]$$

From the coefficient matrix, we obtain the clustering result, and it is shown in Table 9. It is seen that SO_7_ and SO_8_ are attributed to the extraordinary class, SO_4_ and SO_6_ are attributed to normal class, and the rests are attributed to the good class. Thus, we can evaluate not only the sensor selection based on the results, but also every single sensor.

**Figure 6:**
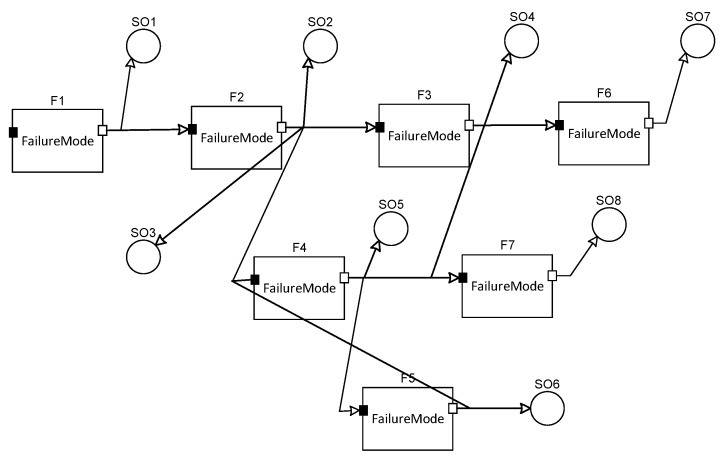
The dependency model of the controller.

To evaluate the different selection methods, we choose the dependency model and GA as the evaluated subject. We construct the dependency model of the controller by a software (Testability modeling and analysis system, TMAS), and the model is shown in Figure 6.

By the software analyzing, we obtain the sensor selection result SO = {SO_1_, SO_5_(SO_6_), SO_7_, SO_8_}, and SO_5_ and SO_6_ are the equivalent sensors.

Then, we use the GA to achieve sensor selection, and the GA is commonly utilized and a typical one in the optimization method. Firstly, we construct the optimization problem, which is as follows:

(18) $$\left\{ \begin{array}{l} \min\sum_{i}e_{j}\\ s.t.\\ \gamma_{FD}\geq FDR\\ \gamma_{FDkey}=100\%\\ \gamma_{FI}\geq FIR \end{array}\right.$$

where *e_j_* is the expense of the *jth* sensor, FDR is the required value of thefailure detection rate, and FIR is the required value of tfailure isolation rate.

$\gamma_{FD}$ is the failure mode rate that the chosen sensors can achieve, and it can be expressed as follows:

(19) $$\gamma_{FD}=\frac{\sum_{j=1}^{ch}\lambda_{j}}{\sum_{i=1}^{n}\lambda_{i}}$$

where $\sum_{j=1}^{ch}\lambda_{j}$ is the failure rate sum of the failure modes that can be detected by the chosen sensors, and $\sum_{i=1}^{n}\lambda_{i}$ is the failure rate sum of all the failure modes. Therefore, $\gamma_{FI}$ has the similar expression, and it can be expressed as follows:

(20) $$\gamma_{FI}=\frac{\sum_{j=1}^{is}\lambda_{j}}{\sum_{j=1}^{ch}\lambda_{j}}$$

where $\sum_{j=1}^{is}\lambda_{j}$ is the failure rate sum of the failure modes, which can be isolated by the detection. Then, we use the GA to solve the optimization problem. The initial population size of the GA is determined as 30, the crossover probability is 0.8, the mutation probability is 0.02, and other parameters are set up by software (MATLAB) automatically. Finally, we obtain the selection result SO = {SO_1_, SO_4_, SO_5_, SO_7_}.

The valuation principle is the proportion of good and extraordinary sensors. If the proportions of the good and extraordinary sensors (P_1_) of different methods are the same, the proportions of extraordinary sensors (P_2_) are compared. If the two kinds of proportions are the same, the different selection methods are defined as equivalent. It is seen that the selection result of the dependency model is better than the GA synthetically because P_1_ and P_2_ of the dependency model are more than the GA, and then the dependency model method is recommended. 

The contrast result can be explained in two ways. From the perspective of the algorithm itself, the selection result given by the GA is under an expense constraint, and the constraint reduces the influence of other factors on sensors, resulting in an expense-oriented result. From the perspective of physical meaning, the result difference between the dependency model and the GA is that the former selects SO_8_ and the later selects SO_4_. SO_8_ samples the characteristic signal and SO_4_ obtains the calculation validity test result. From Figure 4, it is seen that the calculation result directly affects the characteristic signal output, which means the characteristic signal output is in the later position in the control chain. Thus, the characteristic signal output can reflect more information. Therefore, the proposed method is effective and can give a comprehensive evaluation result.

**Figure 7:**
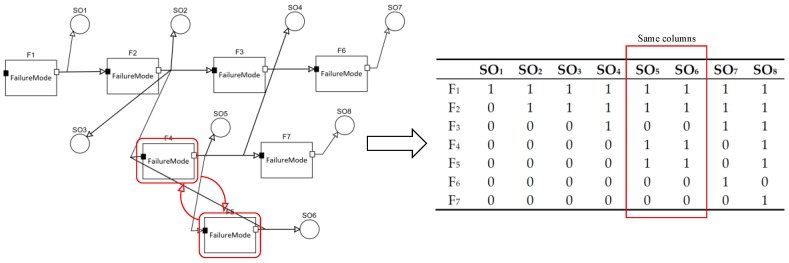
The equivalent sensors.

Moreover, it is noted that SO_5_ and SO_6_ are the equivalent sensors for their same testing response because F_4_ and F_5_ form a closed loop. It can be seen from the dependency model and the dependency matrix as shown in Figure 7. 

In the dependency model theory, SO_5_ or SO_6_ will be chosen equivalently. However, we will choose SO_5_ instead of SO_6_ for the evaluation of SO_5_ (G class) as it is better than SO_6_ (N class) (see Table 9). From the perspective of physical meaning, it is reasonable because SO_5_, the signal sampler, can obtain more useful information and is more sensitive than SO_6_, which is the voltage sensor to monitor the actuator range. Then, the final selection result is SO = {SO_1_, SO_5_, SO_7_, SO_8_}. Therefore, we can improve the selection result to make a more precise choice by adjusting sensors based on the proposed method, which is more convincing and reasonable.

## 4. Conclusions

Sensor selection is a process full of uncertainty, and the selection result usually depends on the expert advice, algorithms, the measured data, and other factors, which brings difficulties to sensor selection and layout. For eliminating or reducing uncertainty, a comprehensive evaluation approach of sensor selection based on grey clustering is proposed. We divide the sensors into three grey classes: extraordinary, good, and normal, and propose three clustering indexes and the combination weight. The indexes are quantified based on a dependency matrix, and the combination weight is determined by considering objective and subjective factors. Based on the proposed method, the key indexes are fully considered, and the different dimensional data is unified, which significantly reduces the uncertainty of sensor selection and gives a standard to evaluate the selection results. An electronic control system of a radar prototype is taken as an example. We use the proposed method to evaluate the effectiveness of different methods of sensor selection, and the evaluation contrast between the dependency model and GA shows that the dependency model gives a better selection result. The evaluation result can be explained from the perspective of an algorithm and physical meaning, which proves the reasoning and effectiveness of the proposed method. Meanwhile, the proposed method is also helpful in improving the selection result by adjusting sensors, which is more reasonable and convincing. Besides that, the proposed method offers the following advantages: (1) This method is good at processing data with different dimensions and is helpful for multidimensional information fusion. (2) This method is simple and easily operated. (3) This method can give a more precise and useful result based on the accurate evaluation of every single sensor. (4) This method is more convincing when one considers objective data and subjective tendency. Therefore, the proposed method can be widely utilized in sensor selection and evaluation for prognostics and health management and be beneficial to the setting and layout of sensors. 

## Figures and Tables

**Table 1:** The selection result of the whitening weight functions.

Index	S_1_	S_2_	S_3_
Detection time	Type Ⅰ	Type Ⅲ	Type Ⅱ
Diagnosis expense	Type Ⅰ	Type Ⅲ	Type Ⅱ
Importance value	Type Ⅱ	Type Ⅲ	Type Ⅰ

**Table 2:** The principle of judgment matrix determination.

a_ij_	Principle
1	*c_i_* is equally important to *c_j_*
3	*c_i_* is weakly important to *c_j_*
5	*c_i_* is apparently important to *c_j_*

**Table 3:** The information about the failure modes and sensors.

Failure Mode		Sensor		
F_1_	Signal acquisition unit fault	SO_1_	Initial input signal	signal sampler
F_2_	Intermediate frequency signal extraction unit fault	SO_2_	Intermediate frequency signal	signal sampler
F_3_	Calculating unit fault	SO_3_	Data acquisition validity test result	voltage sensor
F_4_	Power amplifier unit fault	SO_4_	Calculation validity test result	voltage sensor
F_5_	Actuator unit fault	SO_5_	Power amplifier signal	signal sampler
F_6_	Differential signal extraction unit fault	SO_6_	Actuator range	voltage sensor
F_7_	Characteristic signal output unit fault	SO_7_	Differential signal output manual test result	voltage sensor
-	-	SO_8_	Characteristic signal	signal sampler

**Table 4:** Dependency matrix.

	SO_1_	SO_2_	SO_3_	SO_4_	SO_5_	SO_6_	SO_7_	SO_8_
F_1_	1	1	1	1	1	1	1	1
F_2_	0	1	1	1	1	1	1	1
F_3_	0	0	0	1	0	0	1	1
F_4_	0	0	0	0	1	1	0	1
F_5_	0	0	0	0	1	1	0	1
F_6_	0	0	0	0	0	0	1	0
F_7_	0	0	0	0	0	0	0	1

**Table 5:** Detection time (ms).

	SO_1_	SO_2_	SO_3_	SO_4_	SO_5_	SO_6_	SO_7_	SO_8_
F_1_	185	52	139	82	241	20	266	269
F_2_	-	235	33	294	57	298	165	150
F_3_	-	-	-	204	-	-	132	180
F_4_	-	-	-	-	232	243	-	125
F_5_	-	-	-	-	186	246	-	67
F_6_	-	-	-	-	-	-	69	-
F_7_	-	-	-	-	-	-	-	38

**Table 6:** Diagnosis expense (RMB).

	SO_1_	SO_2_	SO_3_	SO_4_	SO_5_	SO_6_	SO_7_	SO_8_
F_1_	20.7	2.0	23.0	20.2	23.3	10.9	24.3	9.8
F_2_	-	26.4	15.6	19.5	0.1	20.3	15.0	7.7
F_3_	-	-	-	1.5	-	-	29.0	4.9
F_4_	-	-	-	-	8.9	2.5	-	21.3
F_5_	-	-	-	-	24.3	28.4	-	6.2
F_6_	-	-	-	-	-	-	17.8	-
F_7_	-	-	-	-	-	-	-	8.5

**Table 7:** Failure rate (10^−6^/h).

	F_1_	F_2_	F_3_	F_4_	F_5_	F_6_	F_7_
Failure rate	49.9	4.3	27.5	18.2	11.0	0.8	6.8
Key	√	×	√	×	×	×	×

**Table 8:** Whitening weight function.

Whitening Weight Function of Index 1	Whitening Weight Function of Index 2	Whitening Weight Function of Index 3
$\begin{array}{l} f_{1}^{1}=\left\{ \begin{array}{l} 1,x\leq 0.4\\ (0.9-x)\times 2,\\ \begin{array}{cc} & 0.4<x\leq 0.9 \end{array}\\ 0,x>0.9 \end{array}\right.\\ f_{1}^{2}=\left\{ \begin{array}{l} 0,x\leq 0.8\\ \frac{20}{3}(x-0.8),\\ \begin{array}{cc} & 0.8<x\leq 0.95 \end{array}\\ \frac{20}{3}(1.1-x),\\ \begin{array}{cc} & 0.95<x\leq 1.1 \end{array}\\ 0,x>1.1 \end{array}\right.\\ f_{1}^{3}=\left\{ \begin{array}{l} 0,x\leq 1\\ 5(x-1),\\ \begin{array}{cc} & 1<x\leq 1.2 \end{array}\\ 1,x>1.2 \end{array}\right. \end{array}$	$\begin{array}{l} f_{2}^{1}=\left\{ \begin{array}{l} 1,x\leq 0.5\\ (0.9-x)\times 2.5,\\ \begin{array}{cc} & 0.5<x\leq 0.9 \end{array}\\ 0,x>0.9 \end{array}\right.\\ f_{2}^{2}=\left\{ \begin{array}{l} 0,x\leq 0.8\\ \frac{20}{3}\times (x-0.8),\\ \begin{array}{cc} & 0.8<x\leq 0.95 \end{array}\\ \frac{20}{3}\times (1.1-x),\\ \begin{array}{cc} & 0.95<x\leq 1.1 \end{array}\\ 0,x>1.1 \end{array}\right.\\ f_{2}^{3}=\left\{ \begin{array}{l} 0,x\leq 1\\ 5(x-1),\\ \begin{array}{cc} & 1<x\leq 1.2 \end{array}\\ 1,x>1.2 \end{array}\right. \end{array}$	$\begin{array}{l} f_{3}^{1}=\left\{ \begin{array}{l} 0,x\leq 1\\ (x-1)\times 2,\\ \begin{array}{cc} & 1<x\leq 1.5 \end{array}\\ 1,x>1.5 \end{array}\right.\\ f_{3}^{2}=\left\{ \begin{array}{l} 0,x\leq 0.8\\ \frac{20}{3}\times (x-0.8),\\ \begin{array}{cc} & 0.8<x\leq 0.95 \end{array}\\ \frac{20}{3}\times (1.1-x),\\ \begin{array}{cc} & 0.95<x\leq 1.1 \end{array}\\ 0,x>1.1 \end{array}\right.\\ f_{3}^{3}=\left\{ \begin{array}{l} 1,x\leq 0.6\\ 5(0.8-x),\\ \begin{array}{cc} & 0.6<x\leq 0.8 \end{array}\\ 0,x>0.8 \end{array}\right. \end{array}$

**Table 9:** Clustering result.

Sensor	Grey Class	
SO_1_	S_2_	G
SO_2_	S_2_	G
SO _3_	S_2_	G
SO _4_	S_3_	N
SO _5_	S_2_	G
SO _6_	S_3_	N
**SO _7_**	**S_1_**	**E**
**SO _8_**	**S_1_**	**E**

## References

[1] Zhou D.-H., Wei M.-H., Si X.-S. (2014). A Survey on Anomaly Detection, Life Prediction and Maintenance Decision for Industrial Processes. Acta Autom. Sin..

[2] Zeng Z., Wen M., Kang R. (2013). Belief reliability: A new metrics for products’ reliability. Fuzzy Optim. Decis. Mak..

[3] Malinowski M., Adams S., Beling P.A. (2019). Risk Analysis and Prognostics and Health Management for Smart Manufacturing.

[4] Han X., Wang Z., He Y., Zhao Y., Chen Z., Zhou D. (2019). A Mission Reliability-Driven Manufacturing System Health State Evaluation Method Based on Fusion of Operational Data. Sensors.

[5] He Y., Chen Z., Zhao Y., Han X., Zhou D. (2019). Mission Reliability Evaluation for Fuzzy Multistate Manufacturing System Based on an Extended Stochastic Flow Network. IEEE Trans. Reliab..

[6] Xia T., Xi L. (2019). Manufacturing paradigm-oriented PHM methodologies for cyber-physical systems. J. Intell. Manuf..

[7] Xu J., Wang Y., Xu L. (2014). PHM-Oriented Integrated Fusion Prognostics for Aircraft Engines Based on Sensor Data. IEEE Sens. J..

[8] Peng Y., Liu D., Peng X. (2010). A review: Prognostics and health management. J. Electron. Meas. Instrum..

[9] Liu L., Liu D., Zhang Y., Peng Y. (2016). Effective Sensor Selection and Data Anomaly Detection for Condition Monitoring of Aircraft Engines. Sensors.

[10] Yang C., Tian S., Long B., Chen F. (2011). Methods of Handling the Tolerance and Test-Point Selection Problem for Analog-Circuit Fault Diagnosis. IEEE Trans. Instrum. Meas..

[11] Cheng S., Azarian M.H., Pecht M.G. (2010). Sensor Systems for Prognostics and Health Management. Sensors.

[12] Zhu X.H., Li Y.H., Hou S.F. (2013). Sensor optimization placement method for PHM system taking the fault detectability of the sensor into account. J. Astronaut..

[13] Shi L.-Y., Lin X.-G., Shi M. (2012). A key metric and its calculation models for a continuous diagnosis capability base dependency matrix. Metrol. Meas. Syst..

[14] Liu C., Wang N., Wang D. (2013). Fault Diagnosability Analysis Method Based on Dependency Model for Liquid-Floated Gyros. Aerosp. Control Appl..

[15] Chi G. (2014). Optimal Sensor Placement for Model-Based Fault Detectability and Isolability. Ph.D. Thesis.

[16] Yang C., Tian S., Long B. (2009). Test Points Selection for Analog Fault Dictionary Techniques. J. Electron. Test..

[17] Hou W., Yao G., Yan J. A method of testability optimization based on improved particle swarm optimization. Proceedings of the 2014 Prognostics and System Health Management Conference (PHM-2014).

[18] Yang S., Qiu J., Liu G. (2012). Sensor Optimization Selection Model Based on Testability Constraint. Chin. J. Aeronaut..

[19] Yang S.M., Qiu J., Liu G.J., Yang P., Zhang Y. (2013). Testability requirement uncertainty analysis in the sensor selection and optimization model for PHM. J. Phys. Conf. Ser..

[20] Sun J., Sun Q.L., Huang K.L., Xie Y., Li H.R. (2012). Study on Method for Test Points Selection under Uncertainty Based on MBQPSO. Appl. Mech. Mater..

[21] Golonek T., Rutkowski J. (2007). Genetic-Algorithm-Based Method for Optimal Analog Test Points Selection. IEEE Trans. Circuits Syst. II Express Briefs.

[22] Nanda S.J., Panda G. (2014). A survey on nature inspired metaheuristic algorithms for partitional clustering. Swarm Evol. Comput..

[23] Li Shunming C.X. (2008). New Three-State Partition of Fault Classification Method and Its Threshold Confirmation. J. Nanjing Univ. Aeronaut. Astronaut..

[24] Liu Y., Zhao H. (2014). Dynamic information aggregation decision-making methods based on variable precision rough set and grey clustering. Grey Syst. Theory Appl..

[25] Lin C.H., Wu C.H., Huang P.Z. (2009). Grey clustering analysis for incipient fault diagnosis in oil-immersed transformers. Expert Syst. Appl. Int. J..

[26] (1985). Testability Program for Electronic Systems and Equipments.

[27] Yuan Z.J., Sun C.-X., Yuan Z.-Y., Li J., Liao R.-J. (2005). Method of Grey Clustering Decision-making to State Assessment of Power Transformer. J. Chongqing Univ..

[28] Li Y., Chen Y. (2013). Synthetic ranking evaluation of flood disaster based on grey-cloud whitening-weight function. J. Nat. Disasters.

[29] Qu L., Li L., Lee J. (2003). Enhanced diagnostic certainty using information entropy theory. Adv. Eng. Inform..

[30] Wei C.C., Chien C.F., Wang M.J.J. (2005). An AHP-based approach to ERP system selection. Int. J. Prod. Econ..

